# Piceatannol Exerts Anti-Obesity Effects in C57BL/6 Mice through Modulating Adipogenic Proteins and Gut Microbiota

**DOI:** 10.3390/molecules21111419

**Published:** 2016-10-25

**Authors:** Yen-Chen Tung, Yu-Hsuan Lin, Hong-Jhang Chen, Shen-Chieh Chou, An-Chin Cheng, Nagabhushanam Kalyanam, Chi-Tang Ho, Min-Hsiung Pan

**Affiliations:** 1 Institute of Food Sciences and Technology, National Taiwan University, Taipei 10617, Taiwan; emjine@gmail.com (Y.-C.T.); gloomy-trf@hotmail.com (Y.-H.L.); fsthjchen@ntu.edu.tw (H.-J.C.); 2 Department of Biological Science & Technology, China Medical University, Taichung 40402, Taiwan; shenchieh.chou@gmail.com; 3 Department of Tourism, Food and Beverage Management, Chang Jung Christian University, Tainan 71101, Taiwan; anniecheng@mail.cjcu.edu.tw; 4 Sabinsa Corporation, East Windsor, NJ 08520, USA; kalyanam@sabinsa.com; 5 Department of Food Science, Rutgers University, New Brunswick, NJ 08901, USA; ho@aesop.rutgers.edu; 6 Department of Medical Research, China Medical University Hospital, China Medical University, Taichung 40402, Taiwan; 7 Department of Health and Nutrition Biotechnology, Asia University, Taichung 41354, Taiwan

**Keywords:** obesity, piceatannol, high-fat diet, C57BL/6, AMPK, gut microbiota

## Abstract

Obesity is a global health concern. Piceatannol (Pic), an analog of resveratrol (Res), has many reported biological activities. In this study, we investigated the anti-obesity effect of Pic in a high-fat diet (HFD)-induced obese animal model. The results showed that Pic significantly reduced mouse body weight in a dose-dependent manner without affecting food intake. Serum total cholesterol (TC), low-density lipoprotein (LDL), high-density lipoprotein (HDL) levels, and blood glucose (GLU) were significantly lowered in Pic-treated groups. Pic significantly decreased the weight of liver, spleen, perigonadal and retroperitoneal fat compared with the HFD group. Pic significantly reduced the adipocyte cell size of perigonadal fat and decreased the weight of liver. Pic-treated mice showed higher phosphorylated adenosine 5′-monophosphate-activated protein kinase (pAMPK) and phosphorylated acetyl-CoA carboxylase (pACC) protein levels and decreased protein levels of CCAAT/enhancer-binding protein C/EBPα, peroxisome proliferator-activated receptor PPARγ and fatty acid synthase (FAS), resulting in decreased lipid accumulation in adipocytes and the liver. Pic altered the composition of the gut microbiota by increasing Firmicutes and *Lactobacillus* and decreasing Bacteroidetes compared with the HFD group. Collectively, these results suggest that Pic may be a candidate for obesity treatment.

## 1. Introduction

Obesity has become a global health issue. A genetic predisposition toward obesity, a lack of physical activity and increased caloric intake are the primary underlying causes in its development. Excess caloric energy is stored as triacylglycerols (TGs) in adipose tissue, which contribute to the hypertrophy of adipocytes. Both adipocyte hyperplasia and adipocyte hypertrophy contribute to the increase of adipose mass [1]. In addition to fat storage, adipose tissue is itself a major endocrine organ that secretes various adipokines, growth factors, cytokines and hormones involved in energy homeostasis, immunity and insulin sensitivity. Dysfunction of adipose tissue in obese individuals results in the disruption of metabolic homeostasis such as elevated plasma glucose, high serum TGs and high LDL-C levels [2]. Therefore, obesity is highly associated with cardiovascular disease, fatty liver, cancer, type-2 diabetes, stroke and osteoarthritis [3]. Hence, weight reduction and the prevention of obesity is an important field of study for the management of many important and chronic diseases

Adenosine 5′-monophosphate-activated protein kinase (AMPK) is a key enzyme involved in intracellular energy balance and regulates glucose uptake, fatty acid synthesis and oxidation as well as cholesterol and protein synthesis [4]. AMPK is a heterotrimeric protein comprising α, β and γ subunits, where α is the catalytic domain and the β and γ subunits are regulatory domains. When the ratio of AMP/ATP is high, AMPK becomes activated, causing a conformational change of the γ subunit to allow phosphorylation of Thr-172 on the α subunit. AMPK is able to regulate lipid metabolism via multiple pathways. Phosphorylation of AMPK inhibits the peroxisome proliferator-activated receptor PPARγ and the CCAAT/enhancer-binding protein C/EBPα, which are the main transcription factors involved in adipocyte differentiation [5]. Activated AMPK also inhibits downstream acetyl-CoA carboxylase (ACC) activity—the rate-limiting enzyme of de novo fatty acid synthesis—preventing the production of malonyl-CoA from acetyl-CoA. Reduction of intracellular fatty acids reduces the synthesis of TGs and decreases lipid accumulation. Carnitine palmitoyl transferase I (CPT-1) is an important enzyme associated with fatty acid oxidation to increase energy expenditure and it is inhibited by phosphorylated ACC (pACC) and malonyl-CoA [6].

Recent studies have suggested that obesity is correlated with alterations in the gut microbiota. The gut microbiota can be involved in energy balance by affecting the efficiency of harvest of energy from the diet and affect the expression of host genes related to regulation of energy storage and expenditure. In the human gut, the two main bacterial phyla present are Firmicutes and Bacteroidetes. Research has indicated that consuming a high-fat diet increased the proportion of Firmicutes and decreased the proportion of Bacteroidetes and then when the subjects switched to a low-caloric diet and lost weight, the Bacteroidetes percentage increased [7]. Gut microbiota also regulate AMPK activity and were involved in fatty acids oxidation in germ-free mice and conventionalized mice (colonized with the microbiota from the cecum) fed with a high-fat diet (HFD; 41% energy from fat) [8,9]. Therefore, it appears AMPK and gut microbiota play key roles in prevention and treatment of obesity.

Piceatannol (3,3′,4,5′-tetrahydroxy-*trans*-stilbene; Pic) is a natural stilbene found in red wine, grapes, blueberries and passion fruit. It is an analog and metabolite of resveratrol (3,4′,5-trihydroxy-*trans*-stilbene; Res) [10]. Pic has been shown to absorb at higher levels into plasma after oral administration of Pic or Res and has a higher cytotoxicity to human leukemia HL-60 cells than Res [10,11]. Pic also has been shown to decrease TG accumulation by inhibition of the transcription factors C/EBPβ, C/EBPα, and PPARγ in the 3T3-L1 cell model [12]. However, studies on the anti-obesity effect of Pic are still scarce, especially in vivo. Therefore, in this study, we investigated the inhibitory effect of Pic on HFD-induced obesity in C57BL/6 mice and elucidated the mechanism of its anti-obesity effects on AMPK expression and gut microbiota.

## 2. Results

### 2.1. Effect of Pic Supplementation on Body Weight in HFD-Fed C57BL/6 Mice

**Figure 1:**
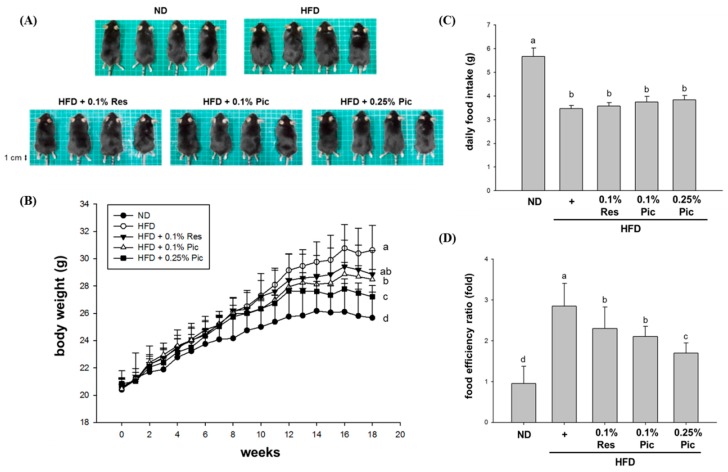
Effect of piceatannol supplementation on body weight in HFD-fed C57BL/6 mice. (**A**) Images of representative mice from each group; (**B**) Weekly body weight measurements over 18 weeks; (**C**) Daily food intake and (**D**) food efficiency ratio. Data are expressed as the means ± SE (*n* = 8 per group). The values with different letters are significantly different (*p* < 0.05) as determined by Duncan’s multiple range test.

In this experiment, five-week-old male C57BL/6 mice with a similar initial weight were randomly assigned into five groups (*n* = 8 per group). The groups consisted of normal diet (ND), HFD, HFD + 0.1% Res, HFD + 0.1% Pic and HFD + 0.25% Pic. After 18 weeks of feeding, the mice were euthanized by CO_2_ asphyxiation for further investigation. Figure 1A shows the morphology of the mice in different groups before sacrifice. Mice fed a HFD were rounder and larger in size compared with the ND group. The 0.25% Pic group had a slimmer appearance than the HFD group, which might be associated with reduced fat accumulation. Regarding the weekly body weight measurements, the HFD group had the highest body weight, significantly greater than the ND group by approximately 5 g (Figure 1B). Although 0.1% Res, 0.1% Pic and 0.25% Pic groups had reduced body weight compared with HFD group, only the 0.25% Pic-treated group showed a significant difference from the HFD group after 18 weeks of ad libitum feeding. There was no significant difference in food intake among the HFD, 0.1% Res, 0.1% Pic and 0.25% Pic groups (Figure 1C), but the food efficiency ratio was significantly reduced from the HFD group in the Res and Pic groups (Figure 1D).

### 2.2. Effect of Pic Supplementation on Adipose Tissue Weight in HFD-Fed C57BL/6 Mice

**Figure 2:**
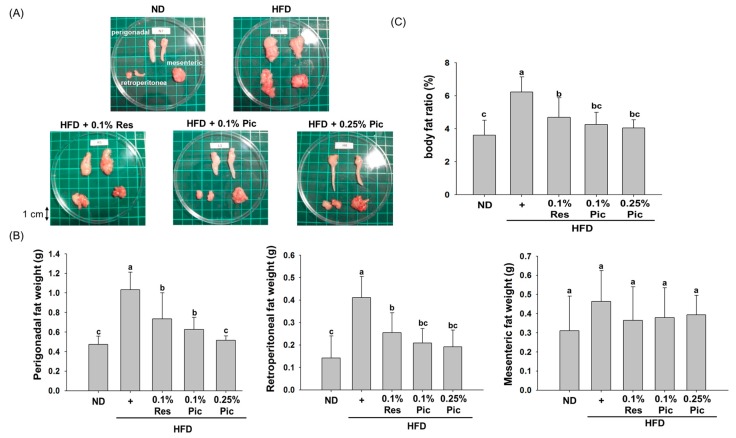
Effect of piceatannol supplementation on adipose tissue weight in HFD-fed C57BL/6 mice. (**A**) Representative images of fat pads from each group; (**B**) The weight of perigonadal, retroperitoneal and mesenteric adipose tissues; (**C**) Body fat ratio, which was calculated using the following formula: adipose tissue weight (perigonadal + retroperitoneal + mesenteric fat weight)/body weight × 100 (%). Data are expressed as the means ± SE (*n* = 8 per group). The values with different letters are significantly different (*p* < 0.05) as determined by Duncan’s multiple range test.

The appearance of perigonadal, retroperitoneal and mesenteric adipose tissue is shown in Figure 2A. The size of the perigonadal and retroperitoneal adipose tissue in the HFD group was larger than the ND group and was concomitant with the higher body weight in the HFD group. Dietary supplementation of Res or Pic decreased the size of the perigonadal and retroperitoneal adipose tissue. As shown in Figure 2B, mice receiving 0.1% Res in the HFD displayed a significant reduction in the weight of the perigonadal adipose tissue. The 0.1% Pic and 0.25% Pic groups significantly decreased the weight of perigonadal adipose tissue in a dose-dependent manner. The same result could be observed in the weight of the retroperitoneal adipose tissue, but there was no dose-dependent effect for the 0.1% and 0.25% Pic treatments. As for the weight of the mesenteric adipose tissue, there was no significant difference among the groups. Both Res- and Pic-treated groups were able to significantly decrease the body fat ratio compared with the HFD group (Figure 2C).

### 2.3. Effect of Pic Supplementation on Adipocyte Size in HFD-Fed C57BL/6 Mice

**Figure 3:**
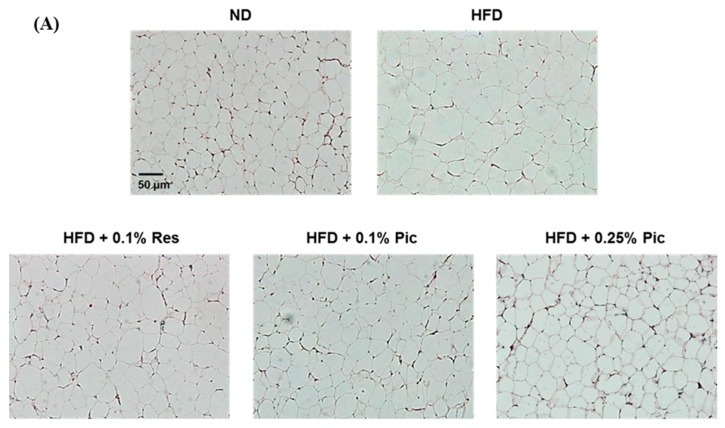

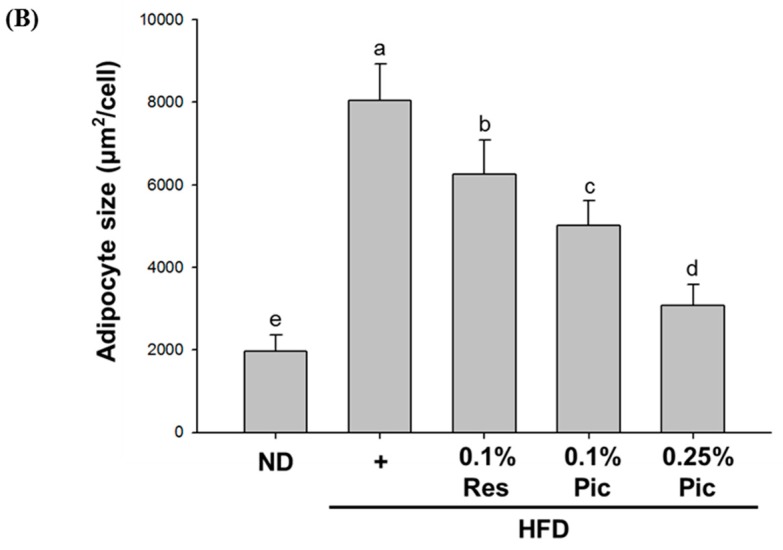
Effect of piceatannol supplementation on adipocyte size in HFD-fed C57BL/6 mice. (**A**) Histology of perigonadal adipose tissues by H&E staining (100× magnification); (**B**) The adipocyte size was quantified microscopically from representative sections (*n* = 15). The values with different letters are significantly different (*p* < 0.05) as determined by Duncan’s multiple range test.

Adipocyte hyperplasia and hypertrophy are underlying causes of obesity. We investigated the effect of Pic on perigonadal adipocyte size by hematoxylin and eosin (H&E) staining (Figure 3A). Adipocyte size in the HFD group was obviously larger in size compared with the ND group, which indicated that consuming extra fat not only increased adipose weight but also the size of the adipocytes. Adipocyte size was significantly reduced in the 0.1% Res, 0.1% Pic, and 0.25% Pic compared with the HFD group.

Figure 3B illustrates the quantified area of perigonadal adipocytes. The adipocyte area of the HFD group was approximately 8000 μm^2^, which was significantly greater than the other groups. Intake of 0.1% Res with a HFD significantly reduced adipocyte area to approximately 6000 μm^2^. The same dosage of Pic reduced the area to 5000 μm^2^, and 0.25% Pic significantly decreased it to 3000 μm^2^, suggesting a dose-dependent effect of Pic on adipocyte size.

### 2.4. Effect of Pic Supplementation on Perigonadal Adipogenic Proteins in HFD-Fed C57BL/6 Mice

Previous results showed that Res and Pic could decrease the weight and adipocyte size of perigonadal fat compared with the HFD group. Therefore, we further investigated the molecular mechanism of anti-obesity effect by Res and Pic in perigonadal adipose tissue by western blot analysis.

**Figure 4:**
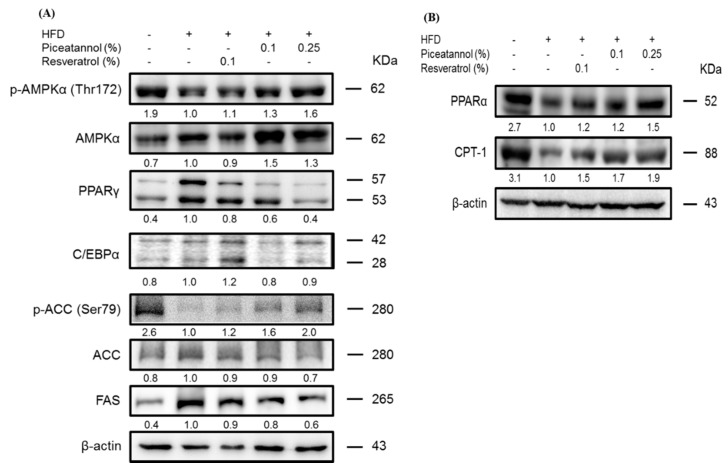
Effect of piceatannol supplementation on (**A**) adipogenic proteins and (**B**) beta-oxidation-related proteins in HFD-fed C57BL/6 mice. Perigonadal protein levels were evaluated by western blot analysis. The values under each lane indicate relative density of the band normalized to β-actin. The western blot is representative of at least three independent experiments.

In the HFD group, protein levels of the adipogenic transcription factors PPARγ and C/EBPα were increased compared to those of the ND group, but these levels, especially PPARγ protein level were suppressed in the 0.25% Pic group (Figure 4A). Activation of AMPKα expression was observed in Pic-treated groups, which up-regulated the expression of pACC. Furthermore, reduced FAS levels were found in both Pic groups compared with the HFD group, suggesting a possible mechanism of Pic-attenuated lipid accumulation in adipocytes. The expression of CPT-1, an enzyme involved in mitochondrial fatty acid oxidation, was significantly increased in both Pic-treated groups compared with the HFD group (Figure 4B). Additionally, the protein expression of PPARα, an upstream transcription factor of CPT-1, was markedly decreased in the HFD group but was increased in both Pic-treated groups.

### 2.5. Effect of Piceatannol Supplementation on Organs in HFD-Fed C57BL/6 Mice

**Figure 5:**
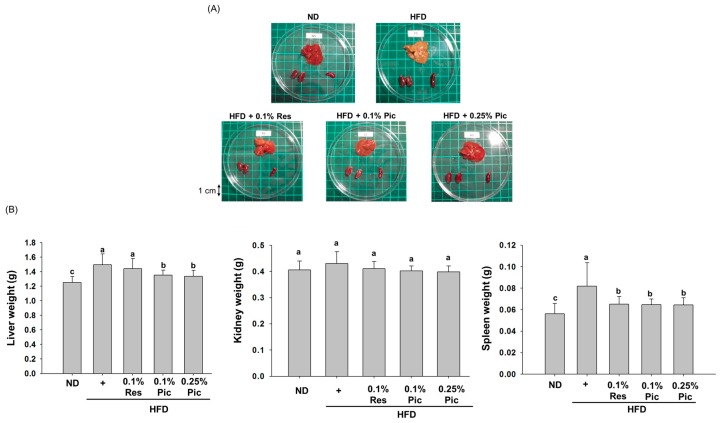
Effect of piceatannol supplementation on organ weight in HFD-fed C57BL/6 mice. (**A**) Morphology of the organs; (**B**) Weights of the liver, kidney and spleen. Data are expressed as the means ± SE (*n* = 8 per group). The values with different letters are significantly different (*p* < 0.05) as determined by Duncan’s multiple range test.

When the body maintains a positive energy balance, adipose tissue dysfunction may develop ectopic fat and deposit it in several organs, such as the liver, pancreas, and heart, and may finally affect their function [13,14]. To assess fat deposition after 18 weeks of treatment, mice in the different groups were euthanized by CO_2_ asphyxiation. The liver, kidney and spleen from each animal were dissected and weighed for comparison (Figure 5A). The livers of mice in the HFD group changed to a yellow-orange color and increased in size, suggesting that long-term intake of fat did induce lipid accumulation. The average weight of livers from mice in the HFD group was significantly heavier than the other groups (Figure 5B). Liver color remained a normal, bright red color in the Pic-treated groups. Furthermore, both Res and Pic had a significantly decreased spleen weight as compared to the HFD group, but the kidney weight was not significantly different compared to the HFD group.

### 2.6. Effect of Pic Supplementation on Hepatic Adipogenic Proteins in HFD-Fed C57BL/6 Mice

**Figure 6:**
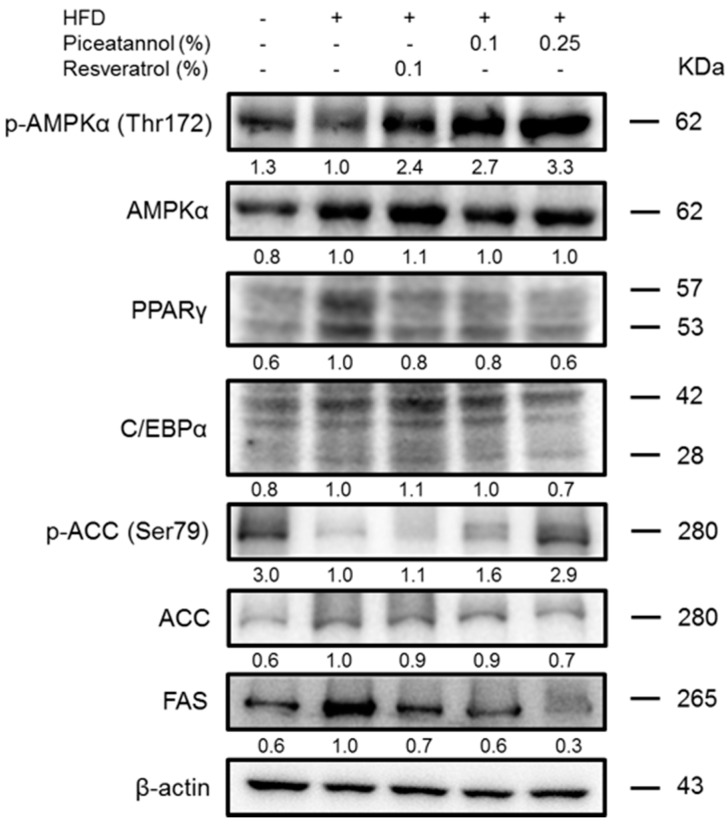
Effect of piceatannol supplementation on hepatic adipogenic protein in HFD-fed C57BL/6 mice. Hepatic adipogenic protein levels were evaluated by western blot analysis. The values under each lane indicated relative density of the band normalized to β-actin. The western blot is representative of at least three independent experiments.

The hepatic adipogenic proteins were subjected to western blot analysis to investigate the molecular mechanism involved in lipid accumulation (Figure 6). The HFD group had higher PPARγ, C/EBPα and FAS protein levels and lower pAMPK and pACC protein levels compared with the ND group. Both Res and Pic seemed to increase pAMPKα and pACC protein level and decrease PPARγ, C/EBPα and FAS compared with the HFD group; this pattern was more evident in the 0.25% Pic group than in the 0.1% Res and 0.1% Pic groups.

### 2.7. Effect of Pic Supplementation on Serum Biochemical Parameters in HFD-Fed C57BL/6 Mice

Sera from each group were analyzed by the National Laboratory Animal Center (Taipei, Taiwan) for various biochemical parameters. HFD consumption significantly raised TC compared with the ND group, but supplementation with Pic significantly suppressed this effect (Table 1).

Plasma TG levels were not significantly different between the groups. The 0.25% Pic group had a significant reduction in LDL-C, HDL-C and the LDL-C/HDL-C ratio compared with the HFD group. Aspartate transaminase (AST) and alanine transaminase (ALT) levels were higher in the HFD group compared with ND group. The 0.25% Pic group had lower ALT and AST level compared with HFD group, however, this difference was not significant. Administration of Pic at either 0.1% or 0.25% was able to significantly attenuate blood glucose (GLU) compared with the HFD group.

### 2.8. Effect of Pic Supplementation on Gut Microbiota in HFD-Fed C57BL/6 Mice

**Figure 7:**
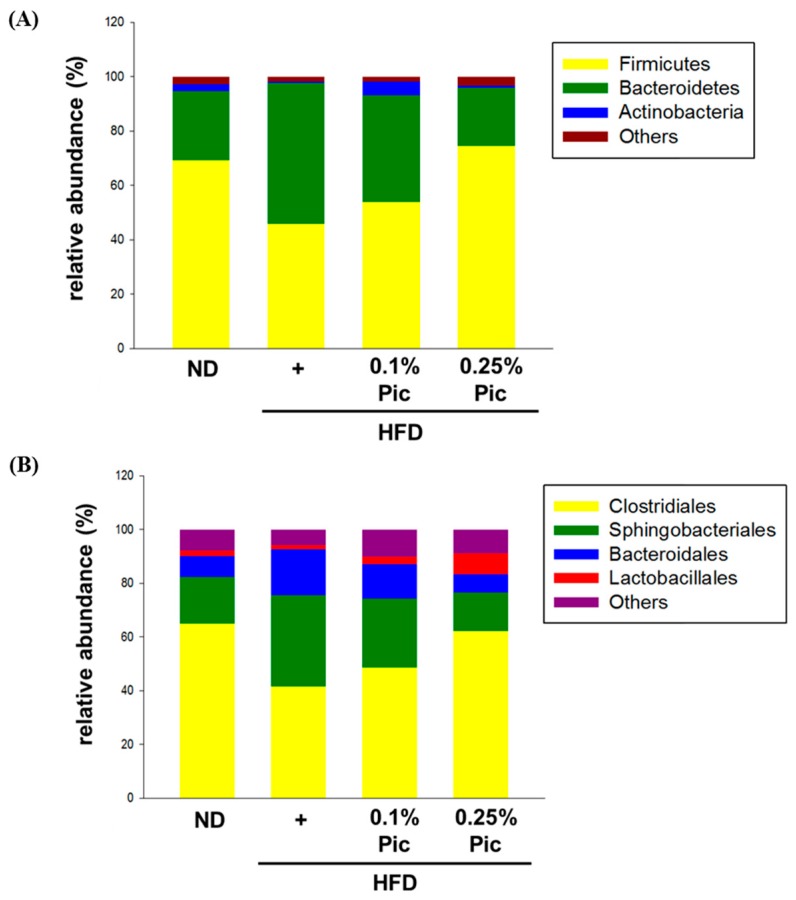
Effect of piceatannol supplementation on the gut microbiota in HFD-fed C57BL/6 mice. (**A**) Phylum classification; (**B**) Order classification. Genomic DNA was extracted from stool samples and identified by the NGS method.

Genomic DNA was extracted from animal stool samples, and gut microbiota were identified by the next generation sequencing (NGS) method. Consumption of a high-fat diet for 18 weeks altered the composition of the gut microbe phyla (Figure 7A and Table 2). The proportion of Firmicute bacteria in the HFD group was 45.84%, which was lower than the 69.17% observed in the ND group. Supplementation with Pic shifted the percentage of Firmicutes to 54.03% and 74.53% in the 0.1% Pic and 0.25% Pic groups, respectively. The other major bacterial phylum in the gut, Bacteroidetes, increased to 51.96% in HFD group about twice as much as in the ND group (25.62%). Intake of Pic reduced Bacteroidetes to 39.24% and 21.37% in the 0.1% Pic and 0.25% Pic groups, respectively. The percentage of *Bifidobacterium* in the gut was relatively low in all groups (Table 2). However, *Lactobacillus* was markedly increased in the 0.1% Pic and 0.25% Pic groups to 2.46% and 7.26%, respectively.

Grouping gut bacteria by order, Clostridiales showed the highest proportion in each group (Figure 7B). Consumption of a high-fat diet decreased its percentage to 41.53%, and supplementation with Pic increased the Clostridiales percentage to 48.56% and 62.34% in the 0.1% Pic and 0.25% Pic groups, respectively. Sphingobacteriales levels were 34.07% in HFD group. Treatment with 0.25% Pic decreased the Sphingobacteriales percentage to 14.29%, closer to the proportion of 17.33% in ND group. Bacteroidales had similar trend as Sphingobacteriales: supplementation with Pic decreased the percentage of Bacteroidales compared with the HFD group. Table 3 highlights the most abundant genera within the Firmicutes and species within the Bacteroidales among each treatment group. The HFD group had higher number of *Pedobacter kwangyangensis* and *Dysgonomonas wimpennyi* from the Bacteroidales, and lower numbers from *Blautia* from the Firmicutes compared with the ND group. Thus, both 0.1% and 0.25% Pic reversed the percentage of gut microbiota compared with HFD group. Moreover, 0.25% Pic showed almost the same percentage of gut microbiota compared with ND group. The result showed that Pic was able to recover the alterations of gut microbiota caused by the HFD.

## 3. Discussion

Obesity is associated with an increased risk for the development of numerous metabolic complications. Many studies indicated that phytochemicals have the potential to inhibit the differentiation of preadipocytes, reduce adipose tissue mass, stimulate lipolysis and induce apoptosis of existing adipocytes [15,16]. Sun et al. showed that administration of 0.1% (*w*/*w*) resveratrol in a 45% HFD for 11 weeks significantly decreased the body weight gain of C57BL/6 mice [17]. However, another study showed 1, 3, 10, or 30 mg/kg body weight of Pic solution did not decrease body weight gain and visceral weights in the mice fed with HFD (56% energy from fat) [18]. In this study, 0.1% Res, 0.1% and 0.25% Pic were able to decrease body weight without altering food intake but only Res did not show a significant difference compared to the HFD group (Figure 1). The reduction of body weight was correlated with decreased adipose tissue weight. Res and Pic significantly decreased perigonadal and retroperitoneal adipose tissue weight (Figure 2B) and decreased the overall body fat ratio compared with the HFD group (Figure 2C). Furthermore, perigonadal adipocyte size was significantly reduced in the Res and Pic groups and Pic showed better effect on adipocyte size than the Res group (Figure 3). AMPK plays a key role in lipid metabolism by affecting transcriptional factors, lipogenesis and fatty oxidation–related proteins [19]. Um et al., showed that 400 mg/kg Res decreased body weight via increased pAMPKα protein expression and increased pACC protein expression compared with the HFD group [20].

Our results showed that pAMPKα was increased and transcription factor PPARγ was decreased upon Pic treatment in perigonadal adipose tissue (Figure 4A). Activation of AMPK by Pic also phosphorylated downstream ACC, which inactivated its function of converting acetyl-CoA to malonyl-CoA and subsequently inhibited the expression of FAS (Figure 4). Alberdi et al. showed that 30 mg/kg Res decreased liver TG accumulation by increased fatty acid oxidation via activated AMPK and increased the expression of CPT-Ia, but Res did not affect the expression of PPARα in rats fed with an obesogenic diet [21]. In the current study, Pic and Res increased the fatty acid oxidation–related protein, PPARα, and CPT-1 protein expression compared with the HFD group within perigonadal fat.

The liver is another organ that regulates lipid metabolism and helps to control energy balance and body weight [22]. A previous study showed that 30 mg/kg Res decreased TG accumulation in the liver by suppressing adipogenesis-related genes such as ACC and PPAR-γ, and SREBP-1 mRNA expression [23]. The current study showed that Pic treatments increased pAMPKα and pACC protein expression and decreased PPARγ, C/EBPα and FAS protein expression compared with the HFD group in the liver (Figure 6B).

Some studies have suggested that a HFD could alter the proportion of phyla in the gut microbiota, specifically, by increasing Firmicutes and decreasing Bacteroidetes [7]. This study showed a reverse trend, in which there was an increase in Bacteroidetes and decrease in Firmicutes in the HFD group (Figure 7A). A previous study showed that 200 mg/kg Res could ameliorate the gut microbiota changes induced by a HFD (50% energy from fat) by increasing the Bacteroidetes-to-Firmicutes ratio and increasing the numbers of *Lactobacillus* and *Bifidobacterium* [24]. Similarly, the same trend was found in a study where Zucker rats (a rat with mutation in the fatty gene (fa) resulted in the obesity) were fed with 45 mg/kg body weight/day, Pic slightly changed the abundance of several *Lactobacillus*, *Clostridium*, and *Bacteroides* species and increased the Bacteroidetes-to-Firmicutes ratios [25]. In this study, 0.25% Pic also markedly increased the numbers of *Lactobacillus* compared with the HFD group. Aside from the change the gut microbiota at the phylum level between obese and normal subjects, more and more studies have showed that the several specific genera and/or species of gut microbiota could play an important role in the metabolism of the host [9]. In this study, Pic had higher numbers of orders Clostridiales and lower Sphingobacteriales in compared with HFD group (Figure 7B). Pic also reversed the percentage of *Blautia* and *P. kwangyangensis* compared with HFD group, suggesting that Pic was able to recover alterations of gut microbiota caused by a high-fat diet.

Although Res has demonstrated many beneficial effects against cancer, inflammation and diabetes [26], its rapid absorption and metabolization affects its oral bioavailability [11]. In contrast, Pic has a higher plasma concentration after oral administration than that of Res in Sprague-Dawley rats and Pic showed better anti-cancer and anti-oxidant properties than Res [11,27]. When Sprague-Dawley rats were given 360 μmol/kg of Res and Pic by stomach intubation, individually, the maximum concentration in plasma for Pic was 8.1 μmol/L and for Res 5.0 μmol/L within 15 min [11]. In this study, both 0.1% Res and Pic decreased body weight, perigonadal fat weight, and adipocyte size, but 0.25% Pic showed better anti-obesity ability than 0.1% Res and Pic in HFD-induced obese animal model, which was perhaps due to the difference of absorption between Res and Pic. Moreover, 0.25% Pic reversed the percentage of gut microbiota compared with HFD group, and the percentage of gut microbiota was almost as same as ND group. Adsorption, distribution in tissue and individual differences could be factors that might affect 0.25% Pic but did not show significant difference between 0.1% Pic. Overall, Pic might exert a more significant anti-obesity effect than Res. In the 0.25% Pic group, based on daily food intake (4 g/per day), and the final body weight (27 g), after unit conversion, 0.25% Pic is around 370 mg/kg body weight/per day. Further use the formulation proposed by Reagan-Shaw et al. at 2007 for dose translation based on body surface area (BSA) to calculate the adult with 60 kg body weight [28]. After calculation, we extrapolated that 0.25% Pic for humans is 30 mg/kg/per day.

## 4. Materials and Methods

### 4.1. Chemicals

Antibodies against FAS, AMPKα, pAMPKα (Thr172), ACC, pACC (Ser79), PPARγ, C/EBPα, PPARα and CPT-1α were purchased from Cell Signaling Technology (Beverly, MA, USA). Mouse β-actin monoclonal antibody was purchased from Sigma Chemical Co. (St. Louis, MO, USA). Cholesterol used in the animal diet was purchased from Hi-Media Laboratories (Mumbai, India). The Bio-Rad protein assay dye reagent was purchased from Bio-Rad Laboratories (Munich, Germany). Western chemiluminescent HRP substrate (ECL), polyvinylidene fluoride (PVDF) membrane, and ammonium persulfate (APS) were purchased from Millipore (Billerica, MA, USA). Xylenes and hematoxylin and eosin stain were purchased from Surgipath (Peterborough, UK). Res and Pic were obtained from Sabinsa Corp. (East Windsor, NJ, USA). The purity of resveratrol and piceatannol was higher than 99% as determined by HPLC.

### 4.2. Animal Experiments

Male C57BL/6 mice (age 5 weeks) were purchased from the National Laboratory Animal Center (NLAC) (Taipei, Taiwan) and housed in a controlled atmosphere (25 ± 1 °C at 50% relative humidity) with a 12 h light/12 h dark cycle. After 1 week of acclimation, animals were randomly distributed into five groups of 8 animals each as follows: ND (15% energy from fat), HFD (45% energy from lard-based fat), and HFD mixed with 0.1% Res, 0.1% Pic or 0.25% Pic (1 kg of HFD contained 1 or 2.5 g Pic powder and mixed thoroughly by a food mixer machine) for 18 weeks. The experimental diets were modified from the Purina 5001 diet (LabDiet, St. Louis, MO, USA), following our previously published experiment [29]. The diet was prepared every week and stored at 4 °C prior to administration. Animals had free access to food and water at all times. Food cups were replenished with fresh chow daily. The diet intake of animals was monitored daily and body weight recorded weekly. All animal experimental protocols used in this study were approved by the Institutional Animal Care and Use Committee of the National Taiwan University (IACUC, NTU). At the end of the study, all animals were euthanized by CO_2_ asphyxiation. Blood samples were collected from the heart for biochemical analysis. The liver, spleen, kidney, and fat pads (perigonadal, retroperitoneal, and mesenteric fat) were immediately removed, weighed, and photographed.

### 4.3. Histopathological Examinations

Perigonadal adipose tissues were dissected, fixed in 10% buffered formalin for at least 24 h, dehydrated with a sequence of ethanol solutions, and processed for embedding in paraffin. Sections of 5–6 μm in thickness were cut, deparaffinized, rehydrated, stained with H&E, subjected to microscopic observation, and imaged. Adipocyte size was determined using a Nikon light microscope (Japan) equipped with an ocular micrometer at 100× magnification in ten random fields per section.

### 4.4. Western Blotting

Liver and perigonadal tissue were homogenized with gold lysis buffer (50 mM Tris-HCl, pH 7.4; 1 mM NaF; 150 mM NaCl; 1 mM EGTA; 1 mM phenylmethanesulfonyl fluoride; 1% NP-40; and 10 μg/mL leupeptin) to extract total protein. The mixture then centrifuged at 10,000× *g* for 30 min at 4 °C. The total protein content was measured by using the Bio-Rad Protein assay. Samples (50 μg of protein) were mixed with 5× sample buffer (0.3 M Tris-HCl (pH 6.8), 25% 2-mercaptoethanol, 12% sodium dodecyl sulfate (SDS), 25 mM EDTA, 20% glycerol, and 0.1% bromophenol blue). The mixtures were boiled at 100 °C for 10 min and subjected to 10% or 6% SDS-polyacrylamide mini-gels at a constant current of 50 mA. Electrophoresis was then performed on these gels. Proteins in the gel were electrotransferred onto an immobile membrane (PVDF membrane) with transfer buffer composed of 25 mM Tris-HCl (pH 8.9), 192 mM glycine, and 20% methanol. The membranes were blocked with blocking solution containing 20 mM Tris-HCl and then immunoblotted with primary antibodies against the target proteins and β-actin. The blots were rinsed with PBST buffer (0.2% Tween 20 in 1× PBS buffer) for 10 min three times. Then, blots were incubated with a 1:5000 dilution of the horseradish peroxidase (HRP)-conjugated secondary antibody and then washed again three times with PBST buffer. The transferred proteins were visualized with an enhanced chemiluminescence detection kit (ECL; Amersham Pharmacia Biotech, Buckinghamshire, UK).

### 4.5. Biochemical Analysis

Plasma samples were separated by centrifugation at 1000× *g* for 15 min. Plasma levels of AST, ALT, TG, TC, LDL, HDL, and GLU were analyzed at the National Laboratory Animal Center, NLAC (Taipei, Taiwan) on a 7080 biochemical analyzer (Hitachi, Tokyo, Japan) according to the manufacturer’s instructions.

### 4.6. Gut Microbiota Classification by Next Generation Sequencing (NGS)

Total DNA was extracted from fresh fecal samples by using the innuSPEED Stool DNA kit (Analytik Jena AG, Jena, Germany) according to the manufacturer’s instructions. The purified DNA was eluted from the spin column, and the concentration was measured by NanoDrop ND-1000 spectrophotometer. The PCR primer sequences from a previous study [30] were used to amplify the 16S rRNA variable region, and the PCR conditions were as follows: 94 °C for 15 min; followed by 20 cycles at 94 °C (20 s), 54 °C (30 s), and 72 °C (60 s); and a final extension at 72 °C for 3 min using an Agena PCR kit (Agena, San Diego, CA, USA). Then, the amplicons were used to construct index-labeled libraries using the Illumina DNA library preparation kit (Illumina, San Diego, CA, USA). We used qPCR (KAPA SYBR FAST qPCR Master Mix) to quantify each library using the Roche LightCycler 480 system then equally pooled the samples to 1 nM before analysis using the Illumina MiniSeq NGS system (Illumina). More than 100,000 reads with paired end sequencing (2 × 150 bp) were generated, and the metagenomics workflow classified organisms from the amplicon using a database of 16S rRNA data (https://basespace.illumina.com). The classification was based on the Greengenes database (https://greengenes.lbl.gov/). The output of the workflow was a classification of reads at several taxonomic levels: kingdom, phylum, class, order, family, genus, and species.

### 4.7. Statistical Analysis

Data are represented as the means ± SE for the indicated number of independently performed experiments. Comparisons of statistical significance between groups were made by one-way analysis of variance and Duncan’s multiple range test. A *p*-value < 0.05 was considered statistically significant.

## 5. Conclusions

In this study, 0.25% Pic demonstrated the best effects in the reduction of body weight via activation of AMPK and decreased lipogenesis in adipose tissue and liver compared to the other treatments. Pic also retained a similar gut microbiota to a ND diet, as opposed to the HFD group. Based on these findings, we concluded that Pic is a potential agent for preventing and treating obesity, non-alcoholic fatty liver disease (NAFLD) and obesity-related diseases as well as recovering the alterations of gut microbiota caused by a high-fat diet. Further research is needed to elucidate the role of Pic in maintaining gut microbiota populations in high fat diets.

## Figures and Tables

**Table 1:** Effect of piceatannol supplementation on serum biochemical parameters in HFD-fed C57BL/6 mice.

	ND	HFD	0.1% Res	0.1% Pic	0.25% Pic
TC (mg/dL)	70.6 ± 7.81 ^c^	193.2 ± 30.26 ^a^	159.9 ± 40.65 ^a^	122.8 ± 27.34 ^b^	130.8 ± 12.65 ^b^
TG (mg/dL)	48.3 ± 5.41 ^a^	48.5 ± 7.11 ^a^	40.4 ± 11.93 ^a^	44.1 ± 13.46 ^a^	42.4 ± 10.29 ^a^
LDL-C (mg/dL)	4.7 ± 1.40 ^c^	42.7 ± 9.61 ^a^	37.9 ± 9.06 ^a^	26.4 ± 9.08 ^b^	23.9 ± 2.59 ^b^
HDL-C (mg/dL)	54.9 ± 7.15 ^c^	143.1 ± 22.03 ^a^	119.8 ± 28.61 ^a^	86.7 ± 18.72 ^b^	99.2 ± 10.73 ^b^
LDL-C/HDL-C	0.08 ± 0.03 ^c^	0.28 ± 0.04 ^a^	0.28 ± 0.06 ^a^	0.28 ± 0.07 ^a^	0.24 ± 0.02 ^b^
AST (U/L)	134.6 ± 57.41 ^a^	177.9 ± 79.05 ^a^	184.6 ± 83.84 ^a^	217.4 ± 73.16 ^a^	139.2 ± 50.62 ^a^
ALT (U/L)	41.2 ± 4.00 ^a^	118.4 ± 120.21 ^a^	41.1 ± 10.30 ^a^	49.1 ± 14.82 ^a^	39.8 ± 7.66 ^a^
GLU (mg/dL)	422.1 ± 44.06 ^a^	431.7 ± 45.42 ^a^	401.9 ± 73.12 ^a^	374.5 ± 45.96 ^b^	361.3 ± 40.14 ^b^

Data are expressed as the means ± SE (*n* = 8 per group). The values with different letters are significantly different (*p* < 0.05) as determined by Duncan’s multiple range test.

**Table 2:** Effect of piceatannol supplementation on gut microbiota in HFD-fed C57BL/6 mice. The main phyla and probiotic genera *Bifidobacterium* and *Lactobacillus* are shown.

Groups	Firmicutes	Bacteroidetes	F/B Ratio	Bifidobacterium	Lactobacillus
ND	69.17%	25.62%	2.70	0.05%	1.58%
HFD	45.84%	51.96%	0.88	0.07%	1.37%
0.1% Pic	54.03%	39.24%	1.38	0.03%	2.46%
0.25% Pic	74.53%	21.37%	3.49	0.04%	7.26%

**Table 3:** The most abundant genera and species of gut microbiota in HFD-fed C57BL/6 mice.

Groups	Kingdom	Phylum	Class	Order	Family	Genus	Species	% Hits
ND	Bacteria	Firmicutes	Clostridia	Clostridiales	Lachnospiraceae	Blautia		31.943
	Bacteria	Bacteroidetes	Sphingobacteria	Sphingobacteriales	Sphingobacteriaceae	Pedobacter	*kwangyangensis*	14.288
	Bacteria	Bacteroidetes	Bacteroidia	Bacteroidales	Porphyromonadaceae	Dysgonomonas	*wimpennyi*	7.236
HFD	Bacteria	Bacteroidetes	Sphingobacteria	Sphingobacteriales	Sphingobacteriaceae	Pedobacter	*kwangyangensis*	30.228
	Bacteria	Firmicutes	Clostridia	Clostridiales	Lachnospiraceae	Blautia		18.596
	Bacteria	Bacteroidetes	Bacteroidia	Bacteroidales	Porphyromonadaceae	Dysgonomonas	*wimpennyi*	15.058
0.1% Pic	Bacteria	Bacteroidetes	Sphingobacteria	Sphingobacteriales	Sphingobacteriaceae	Pedobacter	*kwangyangensis*	23.152
	Bacteria	Firmicutes	Clostridia	Clostridiales	Lachnospiraceae	Blautia		23.114
	Bacteria	Bacteroidetes	Bacteroidia	Bacteroidales	Porphyromonadaceae	Dysgonomonas	*wimpennyi*	11.505
0.25% Pic	Bacteria	Firmicutes	Clostridia	Clostridiales	Lachnospiraceae	Blautia		30.205
	Bacteria	Bacteroidetes	Sphingobacteria	Sphingobacteriales	Sphingobacteriaceae	Pedobacter	*kwangyangensis*	11.638
	Bacteria	Bacteroidetes	Bacteroidia	Bacteroidales	Porphyromonadaceae	Dysgonomonas	*wimpennyi*	5.884

## Abbreviations

The following abbreviations are used in this manuscript:

ACC	acetyl-CoA carboxylase
ALT	alanine transaminase
AMPK	adenosine 5′-monophosphate-activated protein kinase
AST	aspartate transaminase
C/EBPs	CCAAT/enhancer-binding proteins
CPT	carnitine palmitoyltransferase
FAS	fatty acid synthase
FFA	free fatty acid
GLU	glucose
HDL	high density lipoprotein
H&E	hematoxylin and eosin
IRS	insulin receptor substrate
LDL	low density lipoprotein
pAMPK	phosphorylated AMPK
pACC	phosphorylated ACC
Pic	piceatannol
PPARs	peroxisome proliferators activated receptors
Res	resveratrol
TC	total cholesterol
TGs	triacylglycerols
WAT	white adipose tissue
